# Correlation Between Serum Testosterone Levels and Erectile Dysfunction Severity in Pakistani Men Aged 40-65 Years

**DOI:** 10.7759/cureus.93449

**Published:** 2025-09-28

**Authors:** Khalil Ur Rehman, Fazle Manan, Mir Abid Jan

**Affiliations:** 1 Department of Urology, Institute of Kidney Diseases, Hayatabad Medical Complex, Peshawar, Peshawar, PAK; 2 Department of Andro-Urology, Institute of Kidney Diseases, Hayatabad Medical Complex, Peshawar, Peshawar, PAK

**Keywords:** andrology, correlation, cross-sectional study, erectile dysfunction, hypogonadism, iief-5, pakistani men, testosterone

## Abstract

Background

Erectile dysfunction (ED) is a prevalent and multifactorial condition affecting men worldwide, with potential hormonal underpinnings such as testosterone deficiency playing a critical role. Understanding the strength of the association between serum testosterone and ED severity in Pakistani men is essential to inform targeted diagnostic and therapeutic strategies.

Objective

This study aims to evaluate the correlation between serum total testosterone levels and the severity of ED (measured by the International Index of Erectile Function-5 (IIEF-5)) among Pakistani men aged 40-65 years attending a tertiary care outpatient clinic.

Materials and methods

In this cross-sectional analytical study, we enrolled 110 men aged 40-65 years presenting with ED at the Institute of Kidney Diseases of Medical Teaching Institution (MTI) - Hayatabad Medical Complex, Peshawar, between 1 January and 30 June 2025. ED severity was assessed using the validated IIEF-5. In the morning (8-10 AM), fasting blood samples were collected for the measurement of serum total testosterone using a chemiluminescent immunoassay. Spearman’s rank correlation was used to assess the relationship between testosterone levels and IIEF-5 scores. ANOVA (and the complementary Kruskal-Wallis test) were used to evaluate differences in testosterone across ED severity categories.

Results

The mean age was 54.2 ± 6.7 years, and the mean serum testosterone level was 365.4 ± 102.7 ng/dL. Patients with mild, moderate, and severe ED (32.7%, 38.2%, and 29.1% of the sample, respectively) had mean IIEF-5 scores of 20.4, 13.5, and 7.2, and corresponding testosterone levels of 432.8, 362.1, and 278.6 ng/dL, respectively (all p < 0.0001). A strong positive correlation between testosterone and IIEF-5 score was found (Spearman’s ρ = 0.615, p < 0.0001).

Conclusions

Serum total testosterone shows a strong and significant positive correlation with ED severity in this outpatient Pakistani male cohort. These findings support the inclusion of hormonal evaluation in ED workups, particularly when clinical suspicion of hypogonadism exists.

## Introduction

Erectile dysfunction (ED), the persistent inability to attain or maintain an erection sufficient for satisfactory sexual performance, is a prevalent health concern worldwide, particularly affecting middle-aged and older men [1]. Its multifactorial etiology encompasses vascular, neurological, psychological, and hormonal components [2]. Within this complex interplay, testosterone deficiency has received considerable attention as an important biological factor, though it represents only one of several contributors to ED pathophysiology. Testosterone influences libido, penile endothelial function, and nitric oxide-mediated vasodilation, all of which are critical to erectile physiology [3,4].

Globally, the prevalence of ED increases substantially with age; estimates suggest that up to 50% of men aged 40-70 experience some degree of ED, with severity escalating in the upper age brackets [5]. In Pakistan, reliable epidemiological data remain limited; however, extrapolations from regional and urban studies suggest a high burden, likely comparable to neighboring South Asian countries [6]. Reported prevalence rates vary widely, ranging from 15% to 57% among men aged 40 years or older, with higher rates observed in older cohorts [7]. This variation is likely explained by differences in study design, diagnostic criteria, sample sizes, and population characteristics across studies. Given the demographic shift toward an aging population in Pakistan and the rising prevalence of comorbidities such as diabetes mellitus, hypertension, metabolic syndrome, and psychological stressors, understanding the interplay between testosterone levels and ED severity has become increasingly relevant [8].

International studies have documented low serum testosterone in approximately 10-40% of men with ED, depending on age group and comorbid conditions [9]. Lower testosterone levels often correlate with greater symptom burden, including reduced libido, diminished nocturnal erections, and poorer response to phosphodiesterase type 5 inhibitors [10]. In South Asian contexts, the prevalence of hypogonadism among men with ED may be underreported due to limited screening and cultural reticence to discuss sexual health openly [11]. In Pakistan, small-scale hospital-based investigations have reported a notable proportion of men with ED demonstrating subnormal testosterone levels, although robust, age-stratified community-level data are lacking [12].

This gap is especially pronounced in Pakistani men aged 40-65, a demographic simultaneously at risk due to and sensitive to hormonal aging, yet feasibly accessible for outpatient evaluation. Cultural, dietary, and genetic factors specific to Pakistan may influence baseline testosterone levels, metabolic health, and ED prevalence, underscoring the need for localized research rather than extrapolation from Western cohorts. Given the limited region-specific evidence, particularly within Pakistan, this study is designed to bridge critical gaps by examining the relationship between serum testosterone concentrations and the severity of ED among Pakistani men aged 40-65 years. Elucidating this correlation can inform clinical decision-making, guide screening strategies, and potentially support the development of tailored therapeutic interventions, including hormonal evaluation and management. This study aimed to evaluate the correlation between serum testosterone levels and the severity of ED in Pakistani men aged 40-65 years.

## Materials and methods

This was a cross-sectional analytical study designed to assess the correlation between serum testosterone levels and ED severity in Pakistani men aged 40-65 years. The study was conducted at the Department of Urology, Institute of Kidney Diseases, Medical Teaching Institution (MTI) - Hayatabad Medical Complex, Peshawar, over a six-month period, from January 1, 2025, to June 30, 2025. Ethical approval was obtained from the Hospital Research and Ethics Committee of MTI - Hayatabad Medical Complex, Peshawar (approval number: 2022). Written informed consent was obtained from all participants before their inclusion in the study.

The required sample size to detect a correlation between serum total testosterone and the International Index of Erectile Function-5 (IIEF-5) score was calculated using the Fisher z-transformation (two-sided α = 0.05, power = 80%). An expected effect size of r = 0.30 was used, as it represents a moderate correlation in clinical and behavioral sciences and was reported in a comparable study by Sood et al. (2019) [13]. The minimum required sample size was 90, but to allow for 20% exclusions or missing data, the final target sample size was set at 110 participants.

A non-probability consecutive sampling method was employed, as it allowed the inclusion of all eligible and consenting patients presenting during the study period. This approach was selected due to its feasibility within the available timeframe and resources, a practice previously adopted in similar regional and international studies [6,12]. While this method may limit the generalizability of findings, it remains appropriate for exploratory research aimed at identifying clinically meaningful associations and generating hypotheses.

Male patients aged 40-65 years with a clinical diagnosis of ED for at least three months, confirmed by the IIEF-5 questionnaire (a validated tool provided by the British Association of Urological Surgeons [14-16], and those willing to undergo venous blood sampling for serum testosterone measurement were eligible. Patients with a history of androgen replacement therapy within the past six months, with known endocrine disorders (e.g., hypopituitarism, hyperprolactinemia), with severe psychiatric illness or use of medications affecting sexual function (e.g., antidepressants, antipsychotics), and those with chronic debilitating illnesses such as chronic kidney disease or liver cirrhosis were excluded [7,8,10,17,18].

Eligible participants were identified during routine outpatient consultations. After obtaining informed consent, demographic information, medical history, and comorbidities were documented using a structured proforma. The severity of ED was assessed using the validated IIEF-5 questionnaire. Venous blood samples (5 mL) were collected between 8:00 AM and 10:00 AM under aseptic precautions following an overnight fast. To minimize diurnal variation, a single morning sample was obtained for each participant using a chemiluminescent immunoassay. Although repeat confirmatory assays were not performed due to logistical and financial constraints, strict laboratory protocols and uniform sample timing were maintained to reduce variability.

Data were entered and analyzed using SPSS Statistics version 26.0 (IBM Corp., Armonk, NY, USA). Quantitative variables (e.g., age, serum testosterone levels) were expressed as mean ± standard deviation (SD). Categorical variables (ED severity categories) were presented as frequencies and percentages. The correlation between serum testosterone levels and IIEF-5 scores was assessed using Spearman’s rank correlation coefficient, which was chosen because the data did not meet the assumptions of normality required for Pearson’s correlation and because the relationship between testosterone levels and ED severity may not be strictly linear. A p-value < 0.05 was considered statistically significant.

## Results

A total of 110 male participants aged 40-65 years were enrolled in the study. The mean age was 54.2 ± 6.7 years, and the mean BMI was 26.8 ± 3.4 kg/m². Most participants resided in urban areas (65.5%), while 34.5% were from rural regions. Comorbid conditions included diabetes mellitus in 38.2% of participants, hypertension in 44.5%, dyslipidemia in 28.2%, and current smoking in 30.9%. The overall mean serum testosterone level was 365.4 ± 102.7 ng/dL, and the mean IIEF-5 score was 13.8 ± 4.7.

**Table 1:** Baseline characteristics of study participants (n = 110) Data are presented as mean ± SD or n (%). Statistical tests were not applied to baseline variables. SD: standard deviation, BMI: body mass index, IIEF-5: International Index of Erectile Function-5

Variable		Mean ± SD/n (%)
Age (years)		54.2 ± 6.7
BMI (kg/m²)		26.8 ± 3.4
Residence	Urban	72 (65.5%)
	Rural	38 (34.5%)
Comorbidities	Diabetes mellitus	42 (38.2%)
	Hypertension	49 (44.5%)
	Dyslipidemia	31 (28.2%)
	Current smoker	34 (30.9%)
	Mean serum testosterone (ng/dL)	365.4 ± 102.7
	Mean IIEF-5 score	13.8 ± 4.7

When stratified by ED severity, 32.7% of participants had mild ED, 38.2% had moderate ED, and 29.1% had severe ED. The mean IIEF-5 scores for mild, moderate, and severe ED were 20.4 ± 1.5, 13.5 ± 1.6, and 7.2 ± 1.4, respectively. The corresponding mean serum testosterone levels were 432.8 ± 85.6 ng/dL, 362.1 ± 78.4 ng/dL, and 278.6 ± 64.7 ng/dL. Statistical analysis demonstrated highly significant differences among the three ED severity groups for both IIEF-5 scores (p < 0.0001) and serum testosterone levels (p < 0.0001).

**Table 2:** ED severity and mean serum testosterone Data are presented as mean ± SD and n (%). Differences across ED severity categories were tested using one-way ANOVA. p < 0.05 was considered statistically significant. SD: standard deviation, ANOVA: analysis of variance, ED: erectile dysfunction, IIEF-5: International Index of Erectile Function-5

ED severity category	n (%)	Mean IIEF-5 score ± SD	Mean serum testosterone (ng/dL) ± SD
Mild	36 (32.7%)	20.4 ± 1.5	432.8 ± 85.6
Moderate	42 (38.2%)	13.5 ± 1.6	362.1 ± 78.4
Severe	32 (29.1%)	7.2 ± 1.4	278.6 ± 64.7
Test statistic	-	F = 189.4	F = 56.8
p-value	-	<0.0001	<0.0001

**Figure 1:**
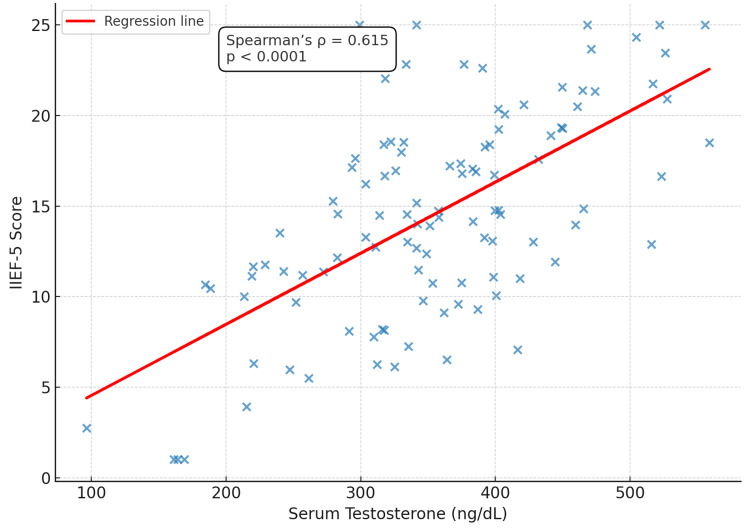
Scatter plot illustrating the correlation between serum testosterone and IIEF-5 scores among study participants (n = 110) Data points represent individual values. A significant positive correlation was observed (Spearman’s ρ = 0.615, p < 0.0001). Statistical significance was set at p < 0.05. IIEF-5: International Index of Erectile Function-5

Correlation analysis demonstrated a statistically significant positive association between serum testosterone levels and IIEF-5 scores (Spearman’s ρ = 0.615, p < 0.0001), indicating that lower testosterone concentrations were strongly linked with greater ED severity. Figure 1 presents this relationship graphically, with each point representing an individual participant (n = 110) and a fitted regression line illustrating the overall trend. Raw values are displayed, and statistical significance was defined as p < 0.05.

## Discussion

In this cross-sectional sample of 110 Pakistani men aged 40-65 years, we observed a strong, statistically significant positive correlation between serum total testosterone and erectile function (IIEF-5), with a Spearman’s ρ of 0.615 and p < 0.0001. Men with more severe ED had progressively lower mean testosterone concentrations (mild: 432.8 ± 85.6 ng/dL; moderate: 362.1 ± 78.4 ng/dL; severe: 278.6 ± 64.7 ng/dL). These findings suggest that androgen status is an important biological contributor to erectile function in middle-aged men. However, as this is an observational, cross-sectional study, the results demonstrate association rather than causation. Our observations are broadly consistent with several recent reports while differing from others; this pattern underscores heterogeneity across populations and study designs.

Several contemporary studies and reviews support these findings. A recent large observational study of men with diabetes and hypertension reported a positive correlation between testosterone concentrations and IIEF-5 scores (ρ ≈ 0.43, p < 0.001), supporting an association between higher testosterone and better erectile function in cardiometabolic cohorts [17]. Likewise, several clinic-based investigations using objective measures (e.g., RigiScan parameters) showed that patients with low testosterone had worse erectile physiology than eugonadal patients, corroborating the testosterone-IIEF link we observed [19].

Systematic reviews and meta-analyses of testosterone replacement therapy (TRT) also provide indirect support. Recent syntheses conclude that TRT can improve sexual function, including erectile outcomes, in men with hypogonadism or low testosterone, though the magnitude of effect is modest and variable across trials. These reviews reinforce the biological plausibility of our observed correlation while highlighting that the relationship is not uniform across all patient subgroups [3,4,20-22].

Conversely, some studies report weaker or absent associations between total testosterone and ED in certain populations. For example, a 2024 community-based study excluding men with hypoandrogenism found no independent association between total testosterone and ED, instead identifying other sex hormones (dehydroepiandrosterone sulfate, estradiol) as more strongly related to erectile function [18]. Similarly, a cross-sectional study from Peshawar in men with type 2 diabetes found no significant association, suggesting that comorbid disease burden (e.g., chronic diabetes, poor glycemic control) may attenuate or obscure the testosterone-ED relationship [6].

Methodological and population differences can explain such inconsistencies. Variability in hormone assays (total vs. free testosterone, assay platform, and timing of sampling), differing inclusion criteria for hypogonadism, and the presence of comorbidities (diabetes, cardiovascular disease, and obesity) all influence the observed associations [1,2,7]. Our study measured morning total testosterone by chemiluminescent immunoassay after an overnight fast, aligning with recommended practice; however, it did not capture free testosterone or sex hormone-binding globulin (SHBG) levels, which can modulate androgen bioactivity. Moreover, clinic-based samples, which are often enriched for metabolic or vascular disease, may yield different patterns than community-based samples. Importantly, as with other cross-sectional designs, our findings demonstrate correlation but not causation. Interventional trials of TRT provide complementary evidence that raising testosterone can improve sexual function in selected hypogonadal men, but responses are variable and context-dependent [20,21].

Clinically, our relatively strong correlation (ρ = 0.615) suggests that in symptomatic middle-aged Pakistani men presenting to urology clinics, testosterone assessment may be informative as part of the ED evaluation. This aligns with guidelines that recommend hormonal testing in men with features of androgen deficiency or inadequate response to first-line therapy [4,21,22]. At the same time, studies showing weak or absent associations highlight the need for a context-specific approach: routine universal testosterone screening in all men with ED may not be cost-effective or diagnostically useful in populations with low pretest probability of hypogonadism [18]. Thus, testosterone testing should be considered selectively, tailored to the clinical scenario rather than applied indiscriminately.

Strengths and limitations

Strengths of the study include a clearly defined age range, standardized morning sampling for testosterone, and the use of a validated IIEF-5 instrument. These elements enhance methodological rigor and ensure greater reliability of the observed association between testosterone levels and erectile function. Limitations include the cross-sectional design, single-center setting, reliance on total rather than free testosterone, and the possibility of residual confounding from unmeasured factors such as depression, medication use, or SHBG levels. Future studies should incorporate free testosterone and SHBG assessments, employ longitudinal designs to establish temporality, and evaluate the effect of interventions such as lifestyle modification, diabetes control, or TRT on erectile outcomes in Pakistani populations.

## Conclusions

Men with more severe ED exhibited notably lower testosterone concentrations, and the strength of the association (ρ = 0.615, p < 0.0001) suggests that hormonal evaluation may be clinically informative in this group. These results underscore the potential value of incorporating testosterone screening as part of the ED assessment in similar outpatient populations, while reinforcing the need to consider individual comorbidity profiles and hypogonadal status. Future longitudinal and interventional studies are warranted to clarify causality and to better define the role of testosterone optimization in improving outcomes among men with ED. Looking ahead, integrating hormonal profiling with broader preventive strategies, such as cardiovascular risk assessment, lifestyle modification, and tailored therapeutic interventions, may provide a more comprehensive approach to managing ED and enhancing overall male health.
